# Sex Hormones Selectively Impact the Endocervical Mucosal Microenvironment: Implications for HIV Transmission

**DOI:** 10.1371/journal.pone.0097767

**Published:** 2014-05-15

**Authors:** Diana Goode, Meropi Aravantinou, Sebastian Jarl, Rosaline Truong, Nina Derby, Natalia Guerra-Perez, Jessica Kenney, James Blanchard, Agegnehu Gettie, Melissa Robbiani, Elena Martinelli

**Affiliations:** 1 Center for Biomedical Research, Population Council, New York, New York, United States of America; 2 Tulane National Primate Research Center, Tulane University Sciences Center, Covington, Louisiana, United States of America; 3 Aaron Diamond AIDS Research Center, Rockefeller University, New York, New York, United States of America; New York University, United States of America

## Abstract

Several studies suggest that progesterone and estrogens may affect HIV transmission in different, possibly opposing ways. Nonetheless, a direct comparison of their effects on the mucosal immune system has never been done. We hypothesize that sex hormones might impact the availability of cells and immune factors important in early stages of mucosal transmission, and, in doing so influence the risk of HIV acquisition. To test this hypothesis, we employed 15 ovarectomized rhesus macaques: 5 were treated with Depot Medroxy Progesterone Acetate (DMPA), 6 with 17-β estradiol (E2) and 4 were left untreated. All animals were euthanized 5 weeks after the initiation of hormone treatment, a time post-DMPA injection associated with high susceptibility to SIV infection. We found that DMPA-treated macaques exhibited higher expression of integrin α_4_β_7_ (α_4_β_7_) on CD4^+^ T cells, the gut homing receptor and a marker of cells highly susceptible to HIV, in the endocervix than did the E2-treated animals. In contrast, the frequency of CCR5^+^ CD4^+^ T cells in DMPA-treated macaques was higher than in the E2-treated group in vaginal tissue, but lower in endocervix. α_4_β_7_ expression on dendritic cells (DCs) was higher in the DMPA-treated group in the endocervical tissue, but lower in vaginal tissue and on blood DCs compared with the E2-treated animals. Soluble MAdCAM-1, the α_4_β_7_ ligand, was present in the vaginal fluids of the control and E2-treated groups, but absent in the fluids from DMPA-treated animals. Both hormones modulated the expression and release of inflammatory factors and modified the distribution of sialomucins in the endocervix. In summary, we found that sex hormones profoundly impact mucosal immune factors that are directly implicated in HIV transmission. The effect is particularly significant in the endocervix. This may increase our understanding of the potential hormone-driven modulation of HIV susceptibility and potentially guide contraceptive policies in high-risk settings.

## Introduction

Epidemiological and laboratory studies suggest that hormonal contraception may alter the risk of HIV acquisition [1]. In particular, women using Depot Medroxy-Progesterone Acetate (DMPA), one of the most widely used progesterone-only contraceptives in the world, were found to be significantly more likely to acquire HIV as compared to women not using hormonal contraception [1]–[3]. However, other studies did not find a significant association between the use of hormonal contraception and the risk of HIV acquisition [4], [5]. Thus, it remains unclear if and how exogenous sex hormones influence susceptibility to HIV.

Varying levels of progesterone and estrogens during the menstrual cycle may also determine a “window of vulnerability” for HIV acquisition in women [6]. Notably, high concentrations of progesterone in the luteal phase have been linked to increased susceptibility to SIV infection in a repeated challenge model of vaginal transmission in Pigtail macaques [7].

Elucidating the relationship between exogenous and endogenous hormones and mucosal susceptibility to HIV may not only inform and guide health policy decision-making, but may also increase our understanding of specific host-related factors that augment the chances of an otherwise rare transmission event.

The most prominent effect of sex hormones is an alteration of the physical properties of the female reproductive tract (FRT). The thickness of the vaginal epithelium and mucus composition vary profoundly during the menstrual cycle and are considered major determinants of susceptibility to infection [8]. However, hormones also influence the immune system directly and indirectly, and recent studies have begun to address how they impact the immune cells in the mucosa as well as innate immune defense mechanisms [7]–[10]. The observed increase in susceptibility to SIV infection due to progesterone implants suggests that high levels of progesterone may increase susceptibility to HIV [7], [11]. In contrast, 17-β estradiol (E2) has been associated with reduced susceptibility to SIV infection and it was shown to protect macaques from SIV vaginal transmission [12], [13]. Nonetheless, to date, no direct comparison of the effects of progesterone and estrogens on the FRT has been reported. Elucidating their differential effects, as we set out to do in this study, may provide important information that will aid in assessing the degree to which hormonal contraceptives may impact susceptibility to mucosal transmission of HIV.

It has been suggested that few immunologic characteristics of the FRT parallel those of the gut, where major HIV-mediated immunologic injury occurs [14]. In fact, about half of the CD4^+^ memory T cells in the FRT express various levels of integrin α_4_β_7_ (Fig S1), Expression of α_4_β_7_ marks immune cells that preferentially traffic to the gut lamina propria (LP) and associated lymphoid tissues (GALT) [15], [16]. However, α_4_β_7_^high^ memory CD4^+^ T cells can also participate in immune responses in the FRT [17]–[19]. It is noteworthy that α_4_β_7_^high^ CD4^+^ T cells are highly susceptible to HIV infection and are preferentially infected during the acute phase of SIV infection [20]–[22]. HIV is able to bind to α_4_β_7_, and this interaction impacts the biology of both T and B cells [22]–[24]. Finally, the frequency of α_4_β_7_^high^ CD4^+^ T cells has been correlated with risk of acquisition following rectal SIV transmission [25].

Alterations in the expression of α_4_β_7_ and other adhesion receptors influence the trafficking of immune cells, including CD4^+^ T cells and dendritic cells (DCs), in and out the mucosal sites that are relevant to HIV transmission. Thus the differential expression of those molecules may delineate diverse FRT microenvironments that vary with respect to the frequency of cells susceptible to productive infection by HIV/SIV. Therefore, we set out to study the impact of sex hormones on the expression of α_4_β_7_ and other mucosal homing receptors. We compared different anatomical locations and, in particular, the lower and the upper FRT.

We found that the hormonal treatments altered the expression of α_4_β_7_ and the frequency of α_4_β_7_^+^ immune cells subsets particularly in the upper FRT. They modulated the expression and the release of MAdCAM-1, the primary α_4_β_7_ ligand, as well as other HIV-linked immune markers. Finally, sex hormones modified the expression and localization of sialomucins in the endocervical tissue indicating changes in the mucus composition.

## Results

### Sex hormones modulate the expression of α_4_β_7_ in different tissues

We hypothesized that progesterone and estrogens can modulate the expression of α_4_β_7_ and the relative frequency of α_4_β_7_^+^ subsets and other adhesion receptors involved in the trafficking of immune cells into and out of the mucosa. To test this hypothesis, we administered a single intramuscular injection of 30mg of DMPA to 5 ovariectomized (OVX) rhesus macaques (RMs). We treated 6 additional OVX animals with 0.02 mg/kg of E2 subcutaneously 3 times/week. In addition we included 4 untreated OVX macaques as controls. In the most common non-human primate models of HIV, when high susceptibility to SIV is required, animals are challenged 5 weeks after injection of 30 mg of DMPA, as this is recognized to be the time of highest susceptibility to SIV infection. Thus, we sacrificed the animals 5 weeks after the onset of DMPA or E2 treatment and collected blood, lymph nodes (LNs), vaginal and endocervical tissues.

All DMPA and control animals had undetectable levels of estrogens, while the E2 group had a daily average (across all animals in the group) of 99 pg/ml. This value is in the range of E2 serum concentrations in normally cycling women: from 45 pg/ml during menstruation to 400 pg/ml during ovulation, after which it quickly falls. In contrast, a single dose of 30 mg of DMPA was chosen because it is routinely used in macaque models of vaginal transmission to increase the susceptibility of the animals to SIV and it is considered the equivalent by weight of the human DMPA dose of 300 mg.

DMPA in serum can be detected 30 minutes after injection and its concentration has been reported to plateau around 1 ng/ml for about 3 months [26].

**Figure 1:**
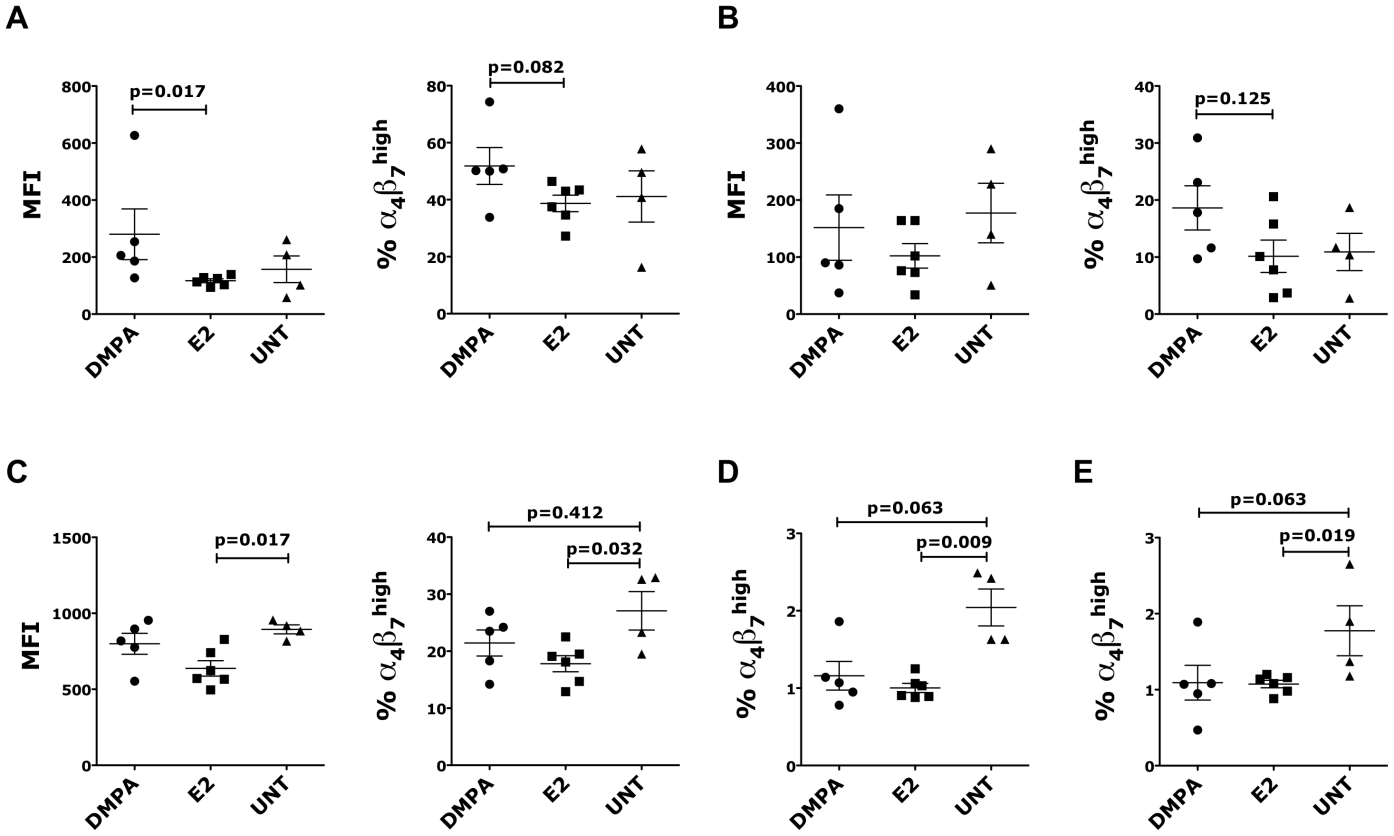
DMPA and E2 modulated α_4_β_7_ expression on CD4^+^ T cells. 15 OVX animals were treated with DMPA (n = 5), E2 (n = 6) or left untreated (UNT, n = 4) and euthanized after 5 weeks from the first injection. Cells from different tissues were separated and the expression of **α_4_β_7_** measured by flow cytometry. Cells were gated on live, singlets, CD3^+^ CD4^+^ cells. (A-C) MFI of **α_4_β_7_** expression (left) and the frequency of **α_4_β_7_** ^high^ cells within the CD95^+^ population (right) are shown for endocervix (A), vaginal (B) tissues and PBMCs (C). (D-E) The frequency of **α_4_β_7_** ^high^ cells within the CD95^+^ population is shown for the MLNs (D) and ILIAC LNs (E). Bars represent mean±SEM. p<0.05 is considered significant and p<0.125 are also shown to indicate a tendency toward a significant difference.

We found that the animals treated with DMPA had a significantly higher expression of α_4_β_7_ on endocervical CD95^+^ CD4^+^ T cells than animals treated with E2 (Fig 1, A left). The frequency of α_4_β_7_^high^ CD4^+^ T cells within the CD95^+^ cells (gating strategy in Fig S1) and of total α_4_β_7_^high^ CD4^+^ T cells was also higher, although the difference was not significant, both in the endocervix and in vaginal tissue (Fig 1, A and B right and data not shown). However, there was no increase in α_4_β_7_ expression on CD4^+^ T cells in the vaginal tissue (Fig 1, B left).

Interestingly, both treatments reduced the frequency of α_4_β_7_^high^ CD4^+^ T cells in blood, mesenteric LNs (MLNs) and iliac LNs (Fig 1 C - E). The reduction was more pronounced in the E2 group than in the DMPA-treated animals. Moreover, in the endocervical and vaginal tissues we examined if DMPA and E2 modulated the expression of CCR6, also expressed on cells highly susceptible to HIV [27], [28] and CD103, the skin homing receptor [29] on CD4^+^ T cells. We found no difference in the expression of CCR6 or CD103 (frequency of positive cells and MFI) in either tissue (Fig S2). Notably, there was no difference in the frequency of CD4^+^ cells within the CD3^+^ population (Fig S2) or as frequency of total live cells (not shown). In blood, iliac and MLN we also measured the expression of CCR9 (gut homing receptor [30]) and CD62L (homing receptor for secondary lymphoid organs [31]). No differences in the expression of CCR6, CD103, CCR9 or CD62L were found (percent positive cells and MFI; not shown).

### The frequency of CCR5^+^ CD4^+^ T cells in the endocervix of the DMPA-treated animals is lower than in E2-treated animals

**Figure 2:**
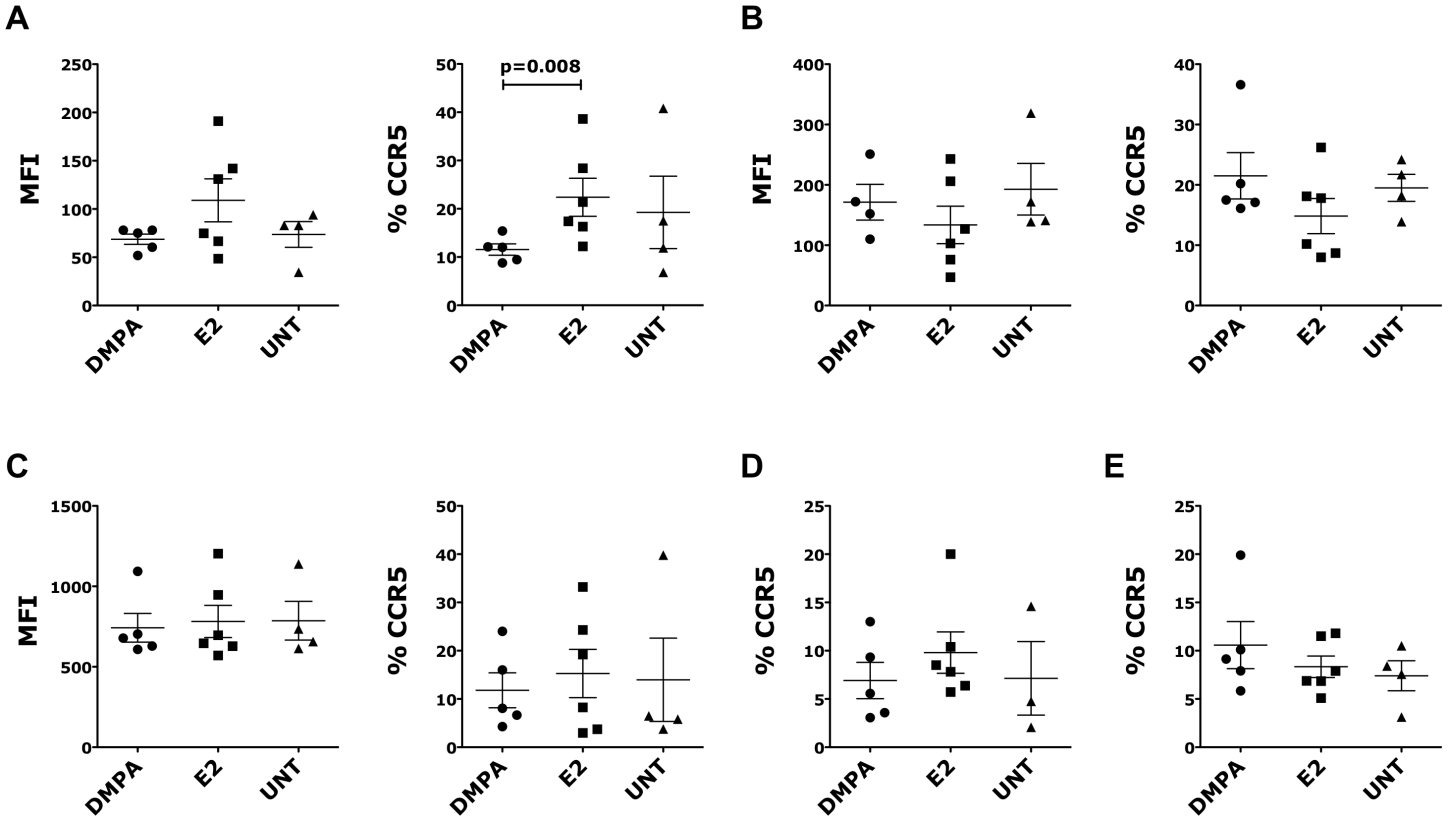
DMPA and E2 modulated CCR5 expression on CD4^+^ T cells. 15 OVX animals were treated with DMPA (n = 5), E2 (n = 6) or left untreated (UNT, n = 4) and euthanized after 5 weeks from the first injection. Cells from different tissues were separated and the expression of **α_4_β_7_** measured by flow cytometry. Cells were gated on live, singlets, CD3^+^ CD4^+^ cells. (A-C) Geometric MFI of CCR5 expression (left) and the frequency of CCR5^+^ cells (right) are shown for endocervix (A), vaginal (B) tissues and PBMCs (C). (D-E) The frequency of CCR5^+^ cells is shown for the MLNs (D) and ILIAC LNs (E). Bars represent mean±SEM. p<0.05 is considered significant and p<0.125 are also shown to indicate a tendency toward a significant difference.

It has been reported that after receiving DMPA, the numbers of CCR5**^+^** immune cells were significantly increased in vaginal tissues in humans, compared to the follicular and/or luteal phases of women not taking hormonal contraception [32]. Moreover, combined oral contraceptive users showed a higher proportion of CCR5^+^ CD4^+^ T lymphocytes compared with combined oral contraceptive non-users [33]. Interestingly, in our study, we found that the frequency of CCR5**^+^** CD4**^+^** T cells (absolute or as frequency of CD95**^+^** cells) in the endocervical tissue was lower in DMPA-treated animals than in E2-treated animals (significant) or in control OVX animals (Fig 2 A). However, in agreement with the previous reports, we found a non-significant increase in the frequency of CCR5**^+^** CD4**^+^** T cells in the vaginal tissue of DMPA-treated animals compared with the E2-treated animals (Fig 2 B). In all the other tissues, the expression of CCR5 and the frequency of CCR5^+^ CD4^+^ T cells were similar in all 3 groups (Fig 2 C-E).

### Sex hormones modulate the expression of α_4_β_7_ and CD80 on DCs

**Figure 3:**
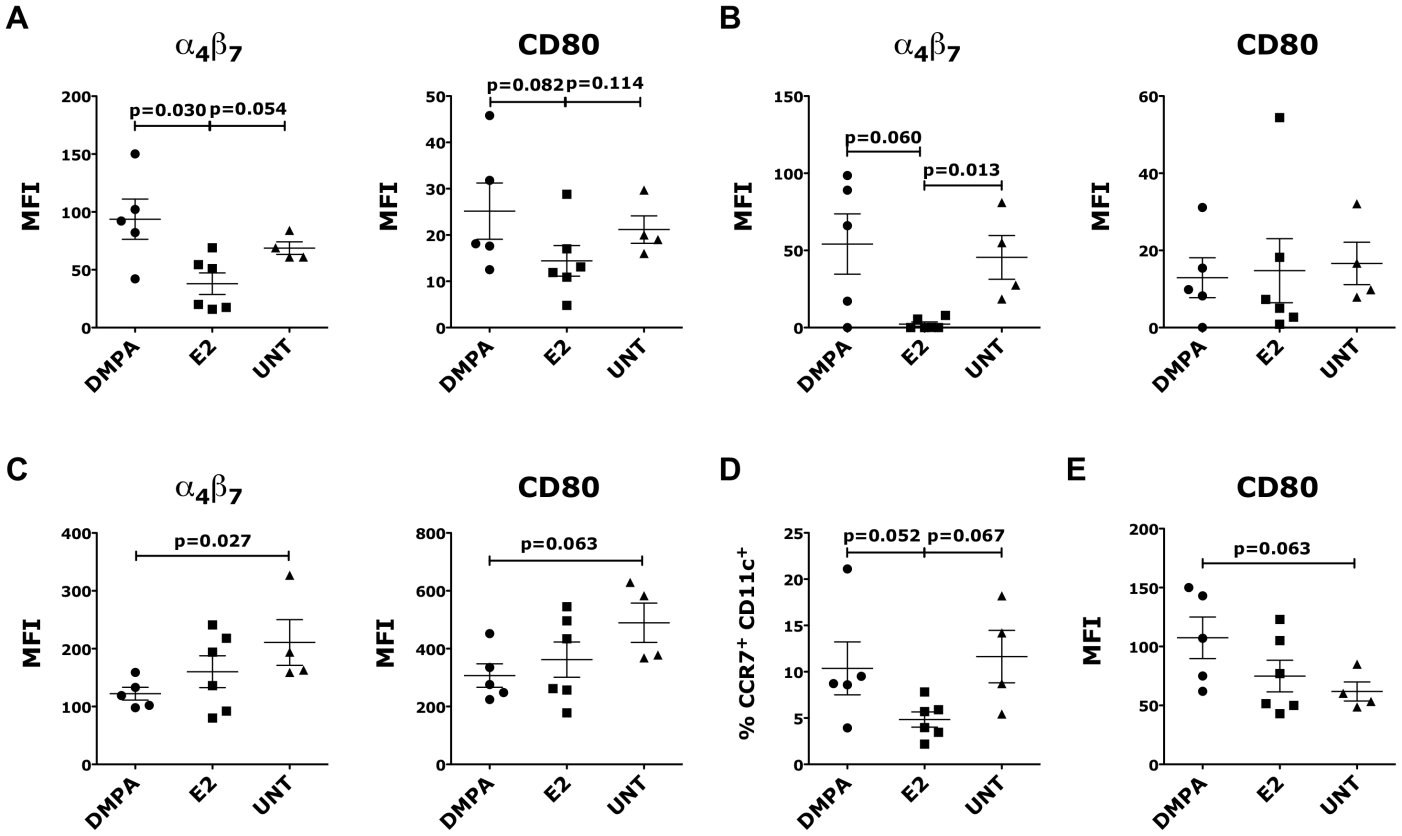
DMPA and E2 modulated α_4_β_7_ and CD80 expression on DCs. Cells isolated from the different tissues were gated on live, singlets, Lin^−^ HLA-DR^+^ A-C) MFI of α_4_β_7_ expression (left) and CD80 (right) are shown for endocervix (A), vaginal (B) tissues and PBMCs (C). (D) The frequency of CCR7^+^ CD11c^+^ DCs in MLN. (E) The MFI of CD80 on DCs in ILIAC LNs is shown. Bars represent mean±SEM. p<0.05 is considered significant. All the results with p<0.125 are also shown to indicate a tendency toward a significant difference.

We found that 5 weeks after a single injection of DMPA and recurrent treatment with E2 there was a significantly lower expression of α_4_β_7_ on Lin^-^ HLA-DR^+^ DCs in the endocervical and vaginal tissues of the E2-treated animals as compared with DMPA-treated and control animals (Fig 3 A and B left).

The expression of CD80 on Lin^−^ HLA-DR^+^ DCs was increased in the endocervix, but not in the vaginal tissues, of DMPA-treated animals compared with the E2-treated animals (Fig 3 A and B right). However, the vaginal tissue of DMPA-treated animals exhibited a non-significantly higher frequency of DCs (Lin^-^ HLA-DR^+^ cells) than that found in the other two groups (not shown).

In contrast, the expression of α_4_β_7_ and CD80 on blood DCs was lower in the DMPA and E2 groups than in controls, with the largest reduction in the DMPA group (Fig 3 C). In the MLNs there was no difference in the expression of α_4_β_7_ and CD80 among the three groups (not shown). However the frequency of CCR7^+^ CD11c^+^ DCs was lower in the E2-treated group compared to the DMPA-treated and control animals (non-significant; Fig 3 D). In the iliac LNs there was an increased expression of CD80 in the DMPA-treated group (Fig 3 E, not significant), but no change in expression of α_4_β_7_ was observed (not shown).

No difference in the expression of CD103, CD11c and CCR7 was found in circulating blood DCs, iliac LNs or vaginal tissues in either treatment group relative to control animals. However, there was a higher frequency of CD103^+^ DCs in the endocervical tissue of DMPA- treated RMs, but this was not statistically significant (not shown).

### Sex hormones impact the expression of MAdCAM-1, CCL21, CCR5 and CCL4 in the endocervix

MAdCAM-1 is the primary α_4_β_7_ ligand and is expressed on the surface of high endothelial venules (HEV) of the GALT, especially in MLNs and Peyers Patches (PPs). Its over-expression is associated with inflammatory conditions of the gut and liver and it is involved in the pathogenesis of chronic inflammatory gastrointestinal diseases including ulcerative colitis and inflammatory bowel disease [34]–[36]. MAdCAM-1 has also been detected outside the endothelial lineage on follicular DCs, fibroblasts and melanoma cells [36]–[38]. Overall MAdCAM-1 is considered a key player in mediating the infiltration of leukocytes into several mucosal tissues in chronic inflammatory states. Its soluble form has been detected in plasma and other body fluids in healthy donors and, at higher levels, in patients with inflammatory conditions of the gastrointestinal tract [39].

**Figure 4:**
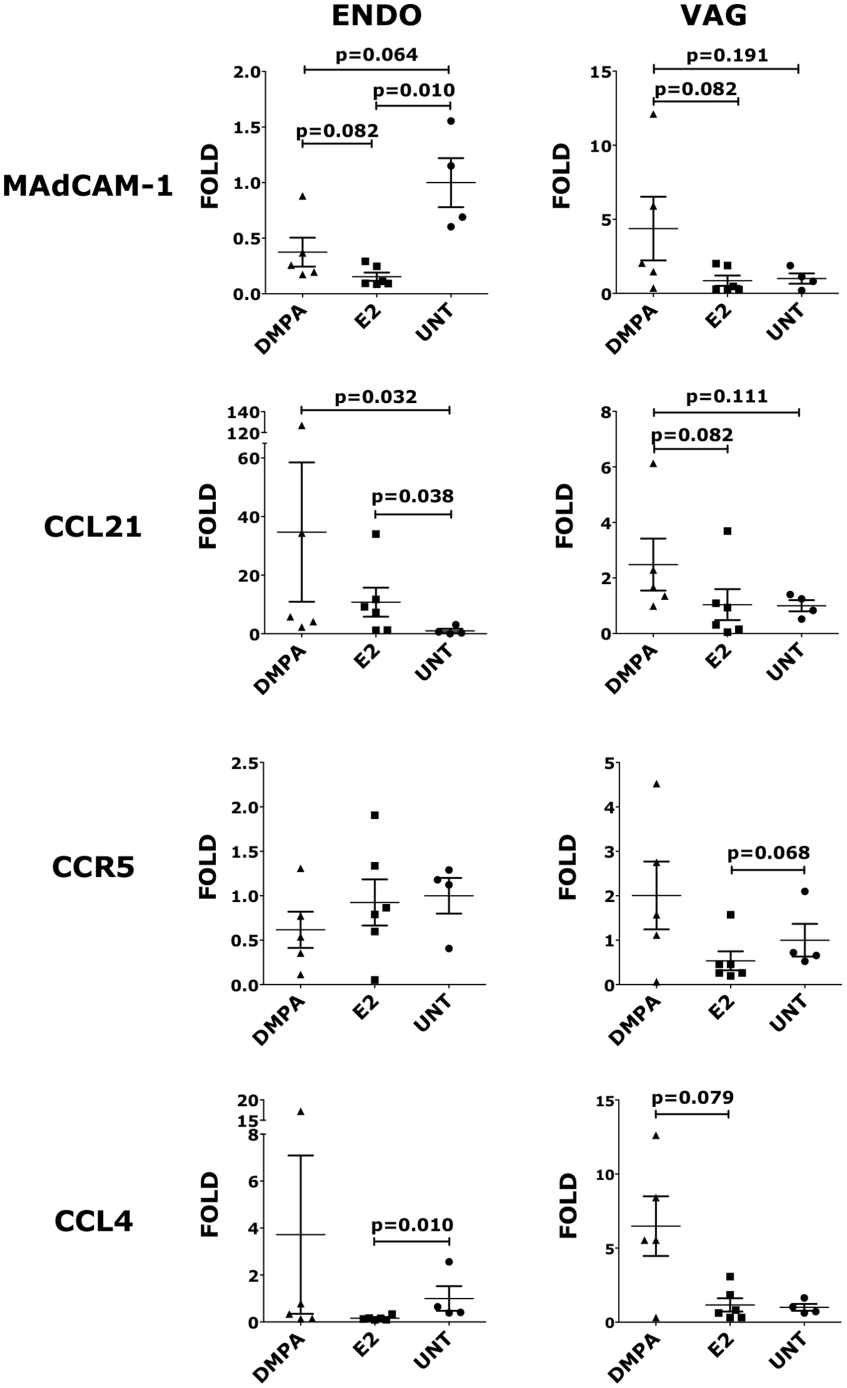
Sex hormones impact the expression of MAdCAM-1, CCL21, CCR5 and CCL4 in the endocervix. Endocervical and vaginal tissues were collected 5 weeks after the initiation of the hormones treatment and RT-qPCR for MAdCAM-1, CCL21, CCR5 and CCL4 was performed using a SybrGreen relative quantification assay. The expression of each gene was calculated relative to one animal chosen for each tissue. The graphs show the fold increase compared to the mean expression of the of the control group (bars represent mean±SEM). p<0.05 is considered significant. All the results with p<0.125 are also shown to indicate a tendency toward a significant difference.

We found that both sex hormones significantly reduced MAdCAM-1 expression in the endocervix, with E2 having a stronger effect (Fig 4, left). In contrast, DMPA increased MAdCAM-1 expression in the vaginal tissue although not significantly (Fig 4, right). E2 and DMPA had no effect on MAdCAM-1 expression in MLNs and iliac LNs (not shown).

CCL21 is another homing molecule key for trafficking of leukocytes, in particular peripheral DCs, to draining LNs. CCL21 triggers T cell arrest by ICAM-1 (CD54), mediates T cell tethering to DCs, and promotes DC adhesion and spread on integrin ligands [40]. Its presence and concentration at the site of HIV exposure may influence not only the immune response, but also the ability of DCs to spread infection. We found that both DMPA and E2 significantly increase CCL21 expression in the endocervix (Fig 4, left), while only DMPA increased CCL21 expression in the vaginal tissue (not significant) (Fig 4, right). Neither hormone affected CCL21 expression in MLNs, iliac LNs or colorectal tissue (not shown).

Differences in the level of CCR5 expression can be detected more accurately by RT-qPCR than by flow cytometry [41]. Nonetheless, even by RT-qPCR we could not detect any significant difference in the expression of CCR5 in vaginal and endocervical tissues, MLNs, or iliac LNs (Fig 4 and not shown). However, E2 significantly reduced expression of the CCR5 ligand, CCL4 in the endocervix, but not in all other tissues evaluated (Fig 4 and not shown).

We also measured the expression of TGFβ and IFNα in all the tissues and there was no significant difference among the three groups.

### Sex hormones influence the levels of soluble inflammatory factors

**Figure 5:**
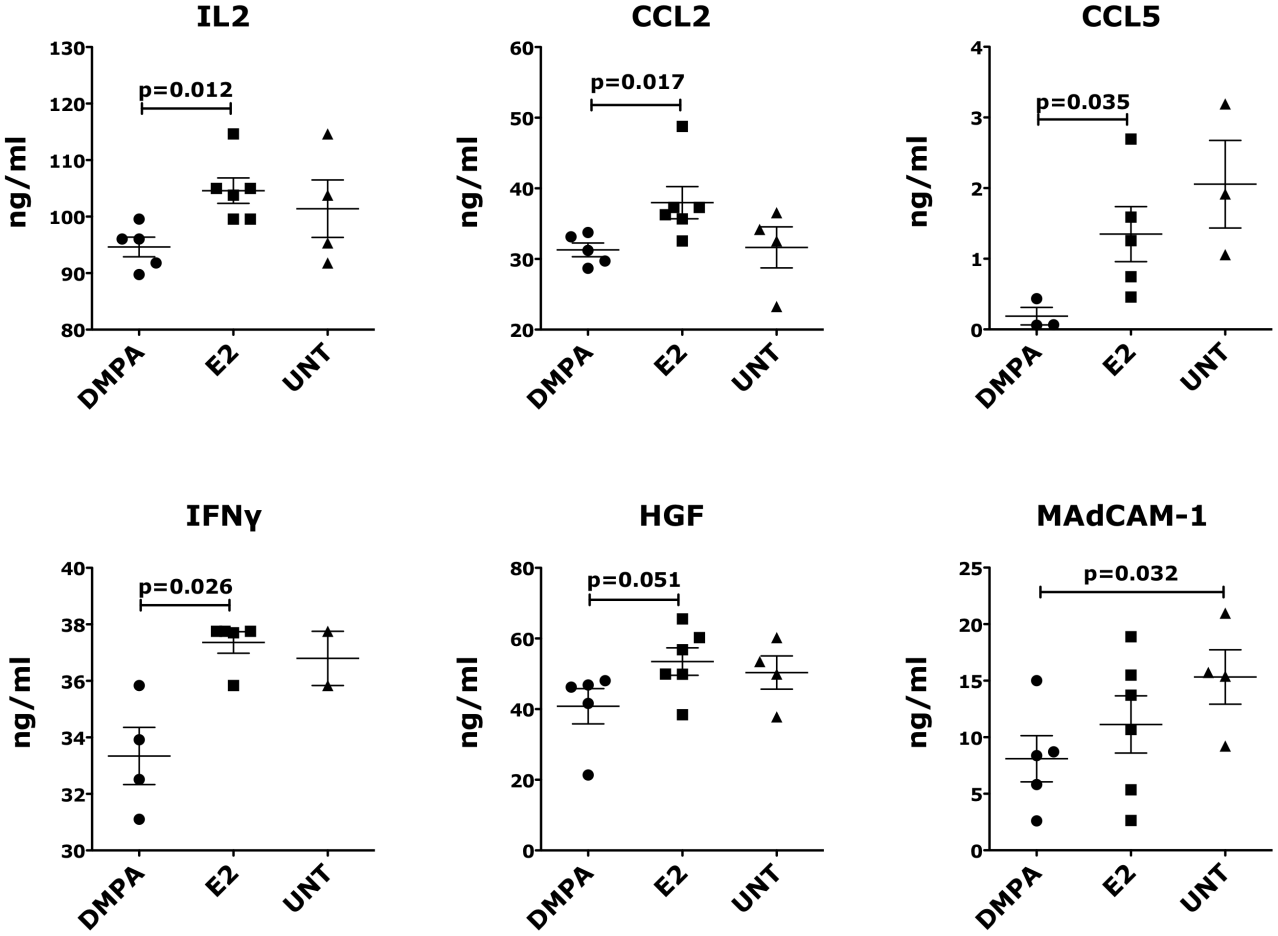
Sex hormones influence the levels of soluble factors in plasma. The concentration of various cytokines and chemokines was measured in plasma of the differently treated (or untreated) animals by 29-Plex Luminex technology and by ELISA (MadCAM-1). Because of technical errors generated by the Luminex machine, we could not measure: CCL5 in 2 DMPA-, 1 E2- and 1 untreated animals; IFNγ in 1 DMPA-treated and 2 untreated animals. Bars represent mean±SEM. p<0.05 is considered significant. All the results with p<0.125 are also shown to indicate a tendency toward a significant difference.

To compare the effect of progesterone and estrogen on an array of soluble inflammatory factors, we measured their concentrations in body fluids at the time of necropsy using a 29-Plex Luminex assay. We found that 5 weeks after DMPA injection, RMs had significantly lower levels of IL2, CCL2 (MCP-1, monocyte chemotactic protein-1), CCL5 (RANTES) and IFNγ in plasma than the E2-treated animals (Fig 5). Also the levels of HGF (Hepatocyte Growth Factor) were lower, although not significantly, in the DMPA-treated compared to the E2-treated animals. The levels of sMAdCAM-1, measured by ELISA, in the DMPA-treated animals were significantly lower than those in the control animals, however there was no significant difference between the DMPA- and E2-treated groups (Fig 5). In contrast, we found no differences in the plasma concentrations of: IL1RA, CXCL11 (I-TAC), MIF Macrophage migration inhibitory factor), FGF-Basic, G-CSF, CCL22 (MDC), IL15, CXCL8 (IL8), EGF, VEGF, CXCL9 (MIG), Eotaxin, CCL4 (MIP-1β), CXCL10 (IP10), GM-CSF, TNFα, IL1β, IL4, IL5, IL6, IL10, IL12, CCL3 and IL17.

**Figure 6:**
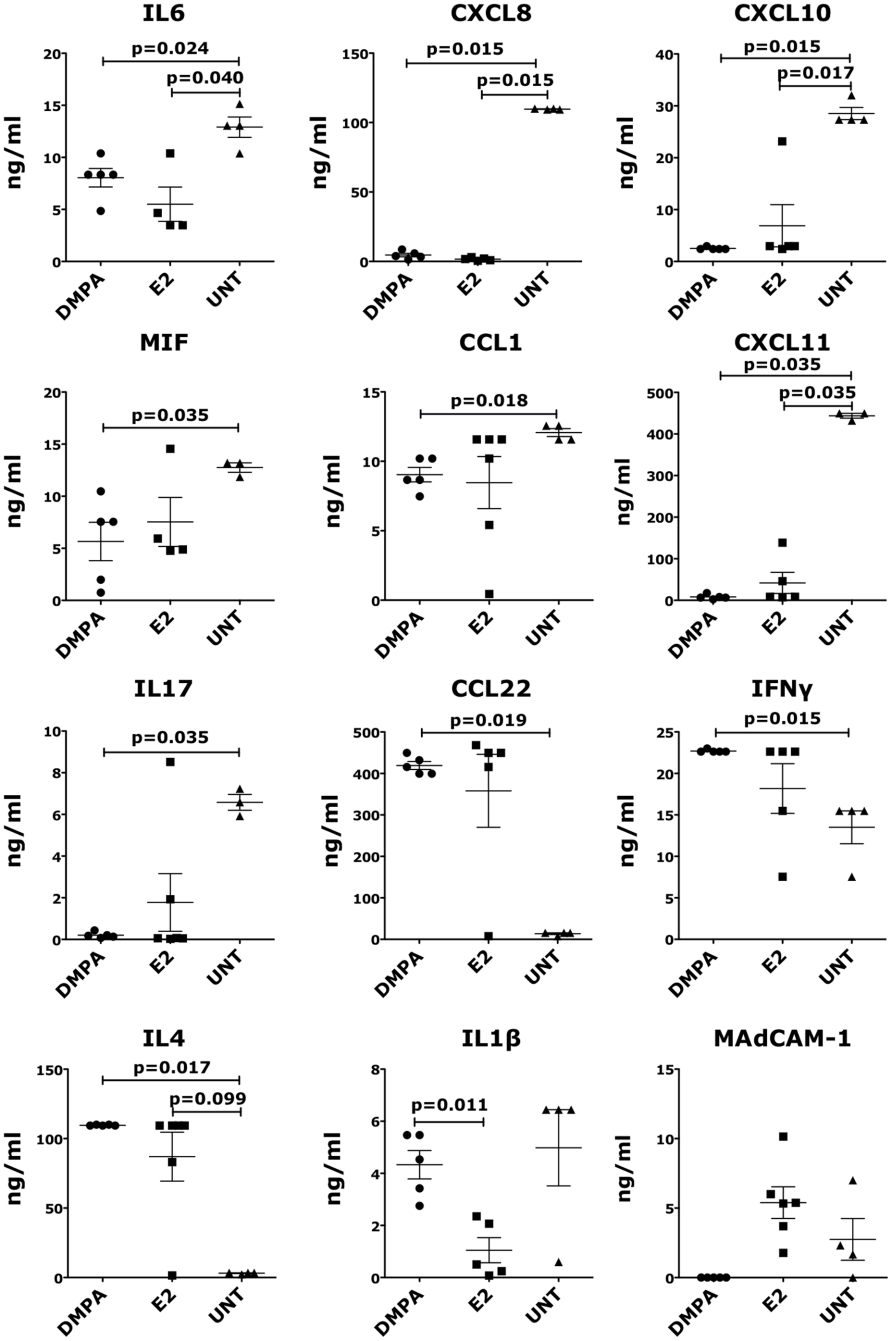
Sex hormones influence the levels of soluble factors in vaginal swabs. The concentration of various cytokines and chemokines was measure in clarified vaginal swabs of animals treated with DMPA-treated, E2-treated or untreated control by Luminex technology. Because of technical errors generated by the Luminex machine, we could not measure: IL6 in 2 E2-treated animals; MIF in 2 E2 treated and 1 untreated animals; CXCL11 in 1 E2- and 1 untreated animals; IL17 in 1 untreated animal, CCL22 and IFNγ in 1 E2 animal. Bars represent mean±SEM. p<0.05 is considered significant and all the results with p<0.125 are also shown to indicate a tendency toward a significant difference.

In the vaginal fluids, the levels of the inflammatory cytokines and chemokines IL6, CXCL8, CXCL10 and CXCL11 were significantly lower in both DMPA and E2 groups than in the controls. MIF, CCL1 and IL17 were significantly lower in the DMPA group compared with the controls (Fig 6). In contrast, the levels of CCL22, IFNγ and IL4 were higher in the DMPA-treated group (significant) and in the E2-treated group (not significant) than in the control group. The concentration of IL1β was significantly lower in the E2 group compared with the DMPA group, while the difference with the untreated group was non-significant probably because of an outlier in the untreated-RMs. Interestingly, no sMAdCAM-1 was detected in the vaginal fluids of the macaques treated with DMPA, while in the E2 group the concentration of sMAdCAM-1 was higher, although not significantly, than in the control group (Fig 6). In contrast, in the vaginal fluids, we found no differences in the concentration of: IL1RA, FGF-Basic, CCL5, HGF, G-CSF, IL15, EGF, VEGF, CXCL9, Eotaxin, CCL4, GM-CSF, TNFα, IL2, IL5, IL10, IL12 and CCL3.

### Sex hormones modulate the distribution of sialomucins in the endocervix

Cervical mucus produced by the endocervix forms a layer that hinders HIV movement and has been implicated in protection from infection by HIV and other sexually transmitted infections (STIs) [42]. The physical character and amount of mucus secreted in the upper FRT changes during the menstrual cycle and the differential expression of mucins by the endocervical epithelium contributes to this all-important physiologic event [43], [44]. Epithelial cells of the human upper FRT express sulfated selectin oligosaccharide ligands (L-selectin ligands) that are a specific type of sialomucin recognized by the mAb MECA-79 [45]–[47]. The subcellular localization of L-selectin ligands plays a key role in endometrial receptivity and its expression varies during different stages of the menstrual cycle [46], [48]. Alteration in sialomucins indicate a differential mucus composition and therefore may be implicated in the ability of the mucus to protect from external pathogens [49].

**Figure 7:**
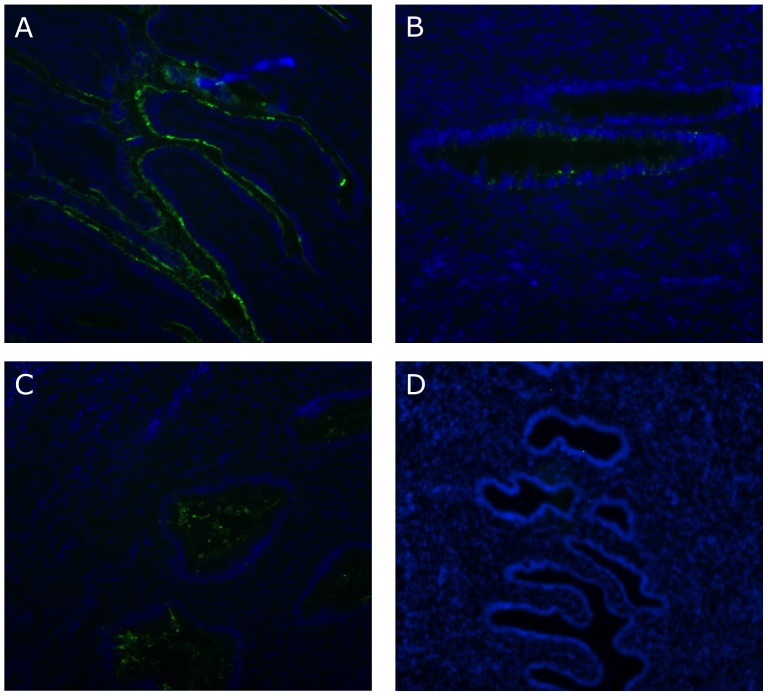
Sex hormones modulate the distribution of sialomucins in the endocervix. Endocervical sections were analyzed for the expression of MECA-79. In (A) no treatment group, MECA-79 staining was significantly stronger with a continuous staining pattern along the glandular epithelium. Both treatment groups showed significantly less staining where the DMPA group (B) had mainly glandular epithelium staining and the E2 group (C) displayed almost exclusively glandular lumenal staining. Isotype control (D) Magnification 20×. One image of at least 5 acquired is shown from one representative animal per group.

We detected a lower reactivity to MECA-79 in the luminal side of the cervical glands in the DMPA- and E2-treated groups compared to controls (Fig 7). Moreover, while the staining in the control group appeared continuous on the surface of epithelial cells (Fig 7 A), the DMPA group displayed a punctate, single-cell staining pattern (Fig 7 B). The staining in the E2 group was more similar to that of the control group, although we also detected some reactivity inside the luminal cavity (Fig 7 C), possibly due to the presence of multiple layers of epithelial cells.

## Discussion

To our knowledge this is the first *in-vivo* study designed to compare the effect of progesterone on mucosal sites involved in HIV transmission, alongside that of estrogens and the absence of sex hormones. Notably, our work focused on immune cell subsets, receptors and immunological markers that have been associated with the likelihood of HIV mucosal transmission and immune cell trafficking.

In cycling macaques, as in humans, there is a continuous variation of the ratio between the levels of progesterone and those of estrogens. To isolate the effect of progesterone from that of estrogen, we used OVX animals. Surgical removal of the ovaries resulted in the termination of the expression and secretion of endogenous sex hormones. This allowed an extensive analysis and comparison of the impact of progesterone (DMPA) and 17-β estradiol in relation to what is observed in the absence of sex hormones (untreated controls). Although our models did not aim to mimic a “real life” condition in women, we chose treatment doses that would achieve plasma concentrations of DMPA similar to those present in women taking DMPA and the E2 average concentrations in serum in normally cycling women. In this way we confidently extrapolated the possible effect of these hormones in humans from the data acquired in our macaque models.

One of the most notable findings of our study is that DMPA and E2 exert their greatest impacts on the region of the FRT corresponding to the endocervix, where more profound differences were noted than in the vaginal tissue. This is important because the endocervix and in particular the transformation zone between the endo and ectocervix have the highest concentrations of monocytes and lymphocytes that are targets for HIV [50]. Moreover, while the lower FRT has a mechanical barrier constituted by a multi-layered squamous epithelium, the endocervix and upper FRT are protected only by a thin single-layered columnar epithelium. These factors indicate a critical increased vulnerability to HIV infection in the endocervix. Indeed, detailed studies of acute SIV infection in rhesus macaques revealed that the rapid expansion of HIV/SIV in tissues likely begins with a small ‘founder’ population of cells in the endocervix [51]. However, one of the major limitations of our study is the low number of animals per group. Many of the differences seen in both endocervical and vaginal tissues that did not achieve significance may become statistically significant with a larger sample size. For this reason we showed and described also results with a p value equal to or lower than 0.125. The low number of animals per group may also explain why most of the significant differences are seen between the DMPA and E2 groups instead than between each of these groups and the untreated animals. Nonetheless, it is of interest that, when the difference with the untreated group is small, the existence of a significant difference between the DMPA-group and the E2-group indicates that the respectively associated changes are in the opposite direction. Additional studies should be performed to investigate the non-significant differences seen in both the upper and lower FRT.

We found that the animals treated with progesterone had higher expression of α_4_β_7_ on CD4^+^ T cells and higher frequency of α_4_β_7_^high^ CD4^+^ T cells than animals treated with E2 and this was particularly significant in the endocervix. The opposite was true for blood, MLN and iliac LNs, where α_4_β_7_, critical for cell homing to mucosal tissues, was expressed at lower levels in the DMPA-treated animals than in the control group. This may be explained by an increased trafficking of α_4_β_7_^high^ CD4^+^ T cells from blood and LNs to the mucosa of the FRT. In contrast, while our results confirm studies reporting that progesterone increases the frequency of CCR5^+^ cells in the vaginal tissue [32], they show that the frequency of CCR5^+^ CD4^+^ T cells in the endocervix is lower in the DMPA-treated animals than in the E2-treated animals. This indicates that sex hormones may have a differential impact in lower and upper FRT.

A profound impact, in particular of E2, was found on α_4_β_7_ expression by DCs in both endocervical and vaginal tissues. DCs are pivotal players in HIV infection. They promote transmission and contribute to the initial viral spread. DCs capture the virus at its portal of entry and transfer it to T cells fueling infection [52]. As is the case for lymphocytes, α_4_β_7_^+^ DCs preferentially traffic to mucosal inductive sites, a critical step in HIV expansion and dissemination after mucosal exposure. We found that E2 decreased the expression of α_4_β_7_ on DCs compared to DMPA (significant) and untreated (non-significant) in the endocervix and, to a lesser extent, in the vaginal tissue. However, we found no corresponding increase in α_4_β_7_^+^ DCs in blood and LNs. This may indicate that the E2 acts locally on mucosal resident DCs decreasing their surface expression of α_4_β_7_. In contrast, DMPA significantly decreased the expression of α_4_β_7_ by blood DCs and this may be linked to a parallel non-significant increase in α_4_β_7_ expression by endocervical DCs.

Overall our findings describe an increased availability of the α_4_β_7_^high^ subset of memory CD4^+^ T cells and α_4_β_7_^+^ DCs at the site of viral exposure, especially in the endocervix. Of note, this was not due to an increased infiltration of CD4^+^ T cells or Lin^-^ HLA-DR^+^ DCs in the tissues. Due to the high susceptibility of α_4_β_7_^high^ CD4^+^ T cells to HIV infection and the ability of α_4_β_7_^+^ DCs to traffic directly to mucosal lymphoid sites, their higher concentration in the endocervix may contribute to the increased in susceptibility linked to high levels of progesterone [1], [7], [11], [53]. Indeed, we previously showed that the frequencies of α_4_β_7_^ high^ CD4^+^ T cells and α_4_β_7_^+^ DCs correlates with susceptibility to rectal SIV infection [25]. In contrast, the lower frequency of these subsets in the E2-treated group compared to the DMPA group may contribute to the protective role of estrogen against SIV acquisition [13].

In line with our results on α_4_β_7_ expression by immune cells, we found that DMPA-treated animals have a non-significant higher expression of the α_4_β_7_ ligand MAdCAM-1 in the vaginal tissue compared with the E2-treated animals. However, puzzling, both hormones had lower MadCAM-1 expression the endocervix compared with untreated animals. MadCAM-1 can be considered a marker of inflammation and the lower expression in the endocervix due to hormonal treatment is in agreement with results showing a lower concentration of inflammatory factors in vaginal fluids of animals treated with either hormone. However, more studies are needed to understand the relationship between α_4_β_7_ expression and MadCAM-1 expression. We also found that both DMPA and E2 treatment increased expression of CCL21 in the endocervix. The lower expression of this key chemokine in OVX macaques appears to be in contrast with data showing an increased immune activation in post-menopausal women [54] and also needs further investigation.

It has been suggested that higher levels of sex hormones and, in particular of progesterone, during the proliferative stage of the menstrual cycle generally suppress the immune system to optimize chances for fertilization and implantation. As consequence, high levels of progesterone during the luteal phase may induce “a window of vulnerability” to HIV infection [6]. In support of this hypothesis, we found that in both DMPA- and E2-treated animals there was a significantly lower concentration of inflammatory factors, including sMAdCAM-1, CXCL8, IL6, CXCL10, CCL1, CXCL11, MIF and IL17. Interestingly, in our model, DMPA appears to induce IFNγ but this does not translate in an increase in IFNγ-induced factors, such as CXCL10 and CXCL11, which are instead decreased by DMPA. These findings need to be confirmed by studies with higher numbers of animals before any possible explanation can be brought forward. In contrast, both hormones induced the release of tolerogenic cytokines such as CCL22, which binds CCR4 on regulatory T cells (Treg) and IL4, which is also produced by inducible Treg cells. This dichotomy in the release of inflammatory and tolerogenic factors is especially evident in vaginal fluids and overall, DMPA had a stronger impact than E2. Intriguingly, the E2-driven decrease in the inflammatory IL1β was absent in the DMPA-treated animals. Taken together, our results indicate that as for progesterone [55], estrogens may also have anti-inflammatory activities. The more tolerogenic environment created by high levels of sex hormones may contribute to dampen the ability of the mucosa environment to fight pathogens such as HIV, delaying the initiation of a potentially protective immune response. Moreover, our results suggest that the combination of high concentrations of both hormones may exert a particularly strong, additive, anti-inflammatory effect in the FRT. This may create a window of *high vulnerability* in the days of the luteal phase when both hormones circulate at higher than average levels. If confirmed, the possibility that a particular combination of progesterone and estrogens is responsible for an increased susceptibility to HIV, rather than high levels of progesterone alone, may have implication for the development and use of combined hormonal contraceptives.

Mucus plays a critical role in the defense of the FRT. The major structural components responsible for its rheological properties are mucins, heavily glycosylated glycoproteins that are secreted by specialized secretory epithelial cells or expressed on their surface [43], [56]. Mucin composition varies with the menstrual cycle and specific combinations of sulfated or non-sulfated, acid or basic sialomucin may be indicative of different inflammatory states in organs such as the gastro intestinal tract and lungs [56]–[59]. The presence of the sulfated epitope of sialomucins recognized by the mAb clone MECA-79 was reported in the endometrium and its expression increases during the proliferative phase of the menstrual cycle in preparation for implantation [45]. Thus detection of differential patterns of MECA-79 staining could indicate variations in one of the most important mechanical barrier against HIV. Our results showed a differential reactivity to MECA-79 in the DMPA and E2 groups compared with the OVX controls. Together with changing its staining pattern, both hormones clearly reduced the levels MECA-79 reactivity, suggesting a decrease in the concentration of mucins in the mucus. This may lessen the density of the vaginal fluids, facilitating access of pathogens, such as HIV to the mucosa. Further investigations are needed to clarify how the differential distribution of mucins on the endocervical epithelium may influence HIV acquisition.

In conclusion, different hormonal environments may alter mucosal tissues swaying the likelihood of a productive transmission event and determining the fate of the virus in the eclipse phase of infection. It was recently reported that, while the risk of SIV acquisition appears to be higher during the luteal phase of the macaque menstrual cycle, infections occurring during the follicular phase give rise to an earlier and higher plasma viral load set point than those in occurring during the luteal phase (Ronald S. Veazey; Non-Human Primate Models of AIDS November 2013). This differential impact on susceptibility and acute phase of infection may influence the course of the disease. Our findings increase our understanding of the individual impact of progesterone and estrogens on the mucosal immune system. We found that they exert a major effect on factors associated with HIV susceptibility especially in the endocervix, the most probable site of HIV penetration and early expansion. Further studies should address how progesterone and estrogen impact each other's effect on the mucosa and to what extent their relative concentrations can contribute to increased risk of HIV acquisition.

## Methods

### Ethics Statement

15 healthy HSV-2 positive adult female Indian rhesus macaques (*Macaca mulatta*, *RM*; mean age: 9 years range: 6–13 years; mean weight: 8.33 kg range: 6.65–12.18 kg) were housed in compliance with the regulations under the Animal Welfare Act, the Guide for the Care and Use of Laboratory Animals, at Tulane National Primate Research Center (TNPRC; Covington, LA). Animals were socially housed, indoors in climate controlled conditions with a 12/12-light/dark cycle. The RMs were monitored continuously by veterinarians to ensure their welfare and were fed commercially prepared monkey chow twice daily. Supplemental foods were provided in the form of fruit, vegetables, and foraging treats as part of the TNPRC environmental enrichment program. Water was available at all times through an automatic watering system. The TNPRC environmental enrichment program is reviewed and approved by the IACUC semiannually. Extensive efforts are made to find compatible pairs for every study group, with additional environmental enrichment of housing space through a variety of food supplements and physical complexity of the environment. A team of 11 behavioral scientists monitors the well-being of the animals and provide direct support to minimize stress during the study period. Veterinarians at the TNPRC Division of Veterinary Medicine have established procedures to minimize pain and distress through several means. Monkeys were anesthetized with ketamine-HCl (10 mg/kg) or tiletamine/zolazepam (6 mg/kg) prior to all procedures. Preemptive and post procedural analgesia (buprenorphine 0.01 mg/kg) was required for procedures that would likely cause more than momentary pain or distress in humans undergoing the same procedures. The above listed anesthetics and analgesics were used to minimize pain or distress associated with this study in accordance with the recommendations of the Weatherall Report. All the animals were euthanized at the end of the study using methods consistent with recommendations of the American Veterinary Medical Association (AVMA) Panel on Euthanasia and per the recommendations of the IACUC. Specifically, the animals were anesthetized with tiletamine/zolazepam (8 mg/kg IM) and given buprenorphine (.01 mg/kg IM) followed by an overdose of pentobarbital sodium. Death was confirmed by auscultation of the heart and pupillary dilation. All studies were approved by the Animal Care and Use Committee of the TNPRC (OLAW assurance #A4499-01) and in compliance with animal care procedures. TNPRC is accredited by the Association for Assessment and Accreditation of Laboratory Animal Care (AAALAC#000594).

### Macaque treatments

The RMs were OVX 4 weeks prior the start of hormone treatment. For the ovariectomy procedure, monkeys were maintained on isoflurane gas anesthesia and the ovaries were visualized and removed via laparoscopy. Monkeys were anesthetized with ketamine-HCl (10 mg/kg) or tiletamine/zolazepam (6 mg/kg) prior to all sampling procedures. Preemptive and post procedural analgesia (buprenorphine 0.01 mg/kg) was used for procedures causing more than momentary pain or distress. 5 RM were treated with a single intramuscular injection of 30 mg of DMPA 5 weeks prior to euthanization, 6 RM were treated with subcutaneous injections of 0.02 mg/kg of E2 (Sigma) in sterile sesame oil 3 times/week for 5 weeks until euthanization [60], [61] and 4 RM were left untreated (controls). After 5 weeks blood samples were obtained, the animals were euthanized and LNs, vaginal and endocervical tissues were collected.

### Cell isolation and flow cytometry

PBMCs were isolated using Ficoll-Hypaque density gradient centrifugation. Iliac and MLNs were cut in small pieces and passed through a 40 µm cell strainer. Vaginal and endocervical biopsies were incubated 45 min in R10 with 1 mg/ml hyaluronidase, 1 mg/ml Collagenase II (Sigma-Aldrich, St Louis, MO) 1 mg/ml DNAseI (Roche, Nutley, NJ). The cell suspension was passed through a 40 µm nylon cell strainer. Cells were stained in PBS with the LIVE/DEAD Aqua dye (Invitrogen) and in PBS 1% FBS 0.1% Sodium Azide with anti-CD4-QDot605 and α_4_β_7_-PE (clone Act1; NHP Reagent Resource, MassBiologics, University of Massachusetts, Boston, MA), the binding of which is not cation dependent. Also included in the T cell panel for blood and LNs anti-: CD3-AF700, CCR9-FITC, CD103-APC, CCR5-PeCy7, CD95-V450, CCR6-DL350 (Biolegend, San Diego, CA), CD62L PCP-Cy5.5, CCR7-APC-Cy7 (Biolegend). DCs anti-: CD3-14-20 (Lin)-V450, HLA-DR BV605, CD80-AF700, CD54-DL350, CD11c-PECy7, CD103-APC (ebioscience, San Diego, CA), CD123-PCPCy5.5; CCR7-AF488. For the biopsies: CD3-AF700, CD4-PCPCy5.5, CD103-APC, CD14-20 V450, α_4_β_7_ PE, CD95 FITC, CCR5 PECy7, CD80 APC-H7, HLA-DR BV6-5, CCR6 DL350. The mAbs in PECy7, PCP-Cy5.5, DL350 and APC-Cy7 were directly conjugated using the Lightning-Link labeling kits (Innova Biosciences, Braham, Cambridge, UK). All the mAbs were from BD Biosciences unless otherwise indicated. At least 200,000 events were acquired in the lymphocyte live-cells gate using the BD LSRII Flow Cytometer and analyzed using FlowJo V9 (TreeStar Inc., Ashland OR).

### RNA isolation and RT-qPCR

RNA was isolated from snap frozen tissues using the RNeasy kit (Qiagen, Limburg, Netherlands), RNA was treated on column with RNase-free DNase (Qiagen) and an additional DNase treatment was performed on the RNA using Ambion DNA-free DNase Treatment and Removal according to the manufacturers' protocols. The RNA was retrotranscribed using the SuperScript VILO cDNA Synthesis Kit (Invitrogen, Life Technologies, Grand Island, NY). Relative qPCR was performed using the SYBR Green PCR Master Mix (Applied Biosystems, Life Technologies). The ViiA7 Real-Time PCR machine (Applied Biosystems) was used for carrying out the reaction. Cycling conditions: 95°C 10 mins, 40 × (95°C 15 sec, 60°C 1 min). Dissociation curves were generated to verify absence of unspecific amplification. Data were analyzed using the ABI Prism 7000 SDS Software (Applied Biosystems). GAPDH was used as endogenous control for sample normalization. All primers were tested for efficiency and compared to the efficiency of the GAPDH reaction. Primers are listed in Table S1. The fold increase in gene expression was measured on one animal chosen as reference and the data were plotted as fold increase on the average of the control animals.

### Soluble factors

The levels of sMAdCAM-1 were measured using the HK337 MAdCAM-1 ELISA kit (Hycult Biotech; detection level 0.4 ng/ml), in plasma (dilution 1∶10) and clarified vaginal swabs (dilution 1∶5) according to the manufacturer's protocol. All the other soluble factors were measured using the monkey Novex multiplex Luminex assay (Cytokine Monkey Magnetic 29-Plex Panel; Invitrogen) on a Luminex 200 machine (Luminex Corporation, Austin, TX). Plasma samples were diluted 1∶20 and swabs 1∶10. Complete list of factors measured: IL1RA, I-TAC, MIF, FGF-Basic, MCP-1, G-CSF, IFNγ, MDC, IL15, CXCL8, EGF, HGF, VEGF, CXCL9, CCL5, Eotaxin, CCL4, CXCL10, GM-CSF, TNFα, IL1β, IL2, IL4, IL5, IL6, IL10, IL12, CCL3, IL17.

### Immunohistochemistry

Formalin-fixed paraffin-embedded tissues were prepared at the TNPRC. Deparaffinization was achieved by incubation 3x in xylene (Fisher Scientific, Pittsburgh, PA) 5 min, 3x in 100% EtOH for 2 min, once in 95% EtOH (Sigma-Aldrich St. Louis, MO) 2 min, once in 80% EtOH for 2 min, 2x in distilled water for 2 min. For antigen retrieval slides were incubated 20 min in 1x Diva Decloaker (Biocare Medical, Concord, CA) at 98°C. Tissues were incubated 40 min in blocking buffer (PBS, 0.2% Fish Skin Gelatin, 10% normal goat serum, 1% BSA), washed in washing buffer (PBS, 0.2% Fish Skin Gelatin, 0.1% Triton X-100) and incubated 1 h at RT in the dark with the antibodies diluted in blocking buffer: 1∶100 AF488 clone MECA-79 and 1∶100 AF568 anti-MAdCAM-1 (clone 314G8; AbDSerotech, Raleigh, NC) or 1∶100 AF568 conjugated Mouse IgG1 Negative Control (AbD Serotec, Raleigh, NC) and Rat IgM Isotype Control (eBioscience, San Diego, CA). Slides were washed 3x with and 4x with PBS, 0.2% Fish Skin Gelatin. ProLong Gold Antifade Reagent with DAPI (Molecular Probes, Grand Island, NY) was used to detect nuclei. Slides were viewed using a wide-field fluorescence microscope (Zeiss, Thornwood, NY) and processed using ImageJ software (NIH, Bethesda, MD).

### Statistics

Mann-Whitney non-parametric test was used to compare variables between groups (DMPA vs E2 vs controls). A two-tailed p = α<0.05 was considered significant. The analysis was performed using Prism5a (GraphPad Software, Inc).

## Supporting Information

Figure S1 **The gating strategy for α_4_β_7_^high^ cells in different tissue**: The frequency of α_4_β_7_^high^ memory CD4^+^ T cells is calculated dividing the frequency of α_4_β_7_^high^ (round gate) by the frequency of all the CD95^+^ cells (naïve untreated RM).
(TIF)

Figure S2 **DMPA and E2 do not modulate the frequencies of CCR6^+^, CD103^+^ and CD4^+^ T cells in vaginal and endocervical tissues**: Cells from endocervical and vaginal tissues were gated on live, singlets and on CD3^+^ CD4^+^ cells (left and center) or on CD3^+^ (right). The frequencies of CCR6^+^ and CD103^+^ cells within CD3^+^ CD4^+^ cells and the frequencies of CD4^+^ within CD3^+^ are shown. Bars represent mean±SEM.
(TIF)

Table S1 **List of primers used for the Sybr Green qPCR measuring the expression of the corresponding genes.**
(DOCX)
