# Supplementary figures and images for: Sex Hormones Selectively Impact the Endocervical Mucosal Microenvironment: Implications for HIV Transmission
Source: PLoS One. 2014 May 15;9(5):e97767. doi: 10.1371/journal.pone.0097767 (PMC4022654; doi:10.1371/journal.pone.0097767)

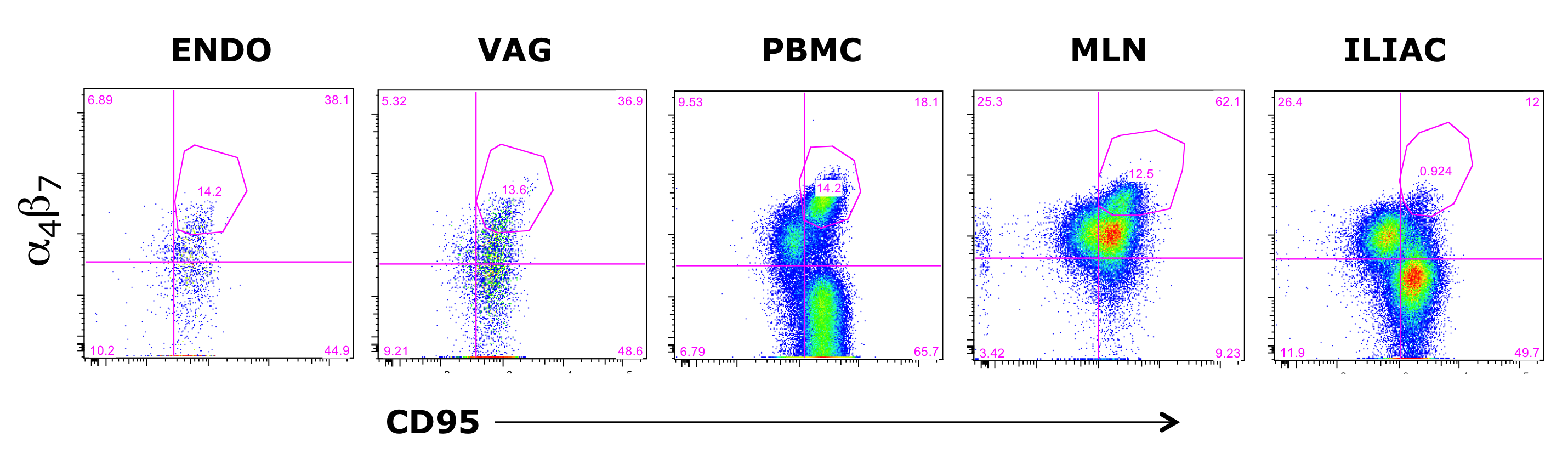

Supplement: Figure S1 — The gating strategy for α4β7high cells in different tissue: The frequency of α4β7 high memory CD4+ T cells is calculated dividing the frequency of α4β7 high (round gate) by the frequency of all the CD95+ cells (naïve untreated RM). (TIF) [file pone.0097767.s001.tif]

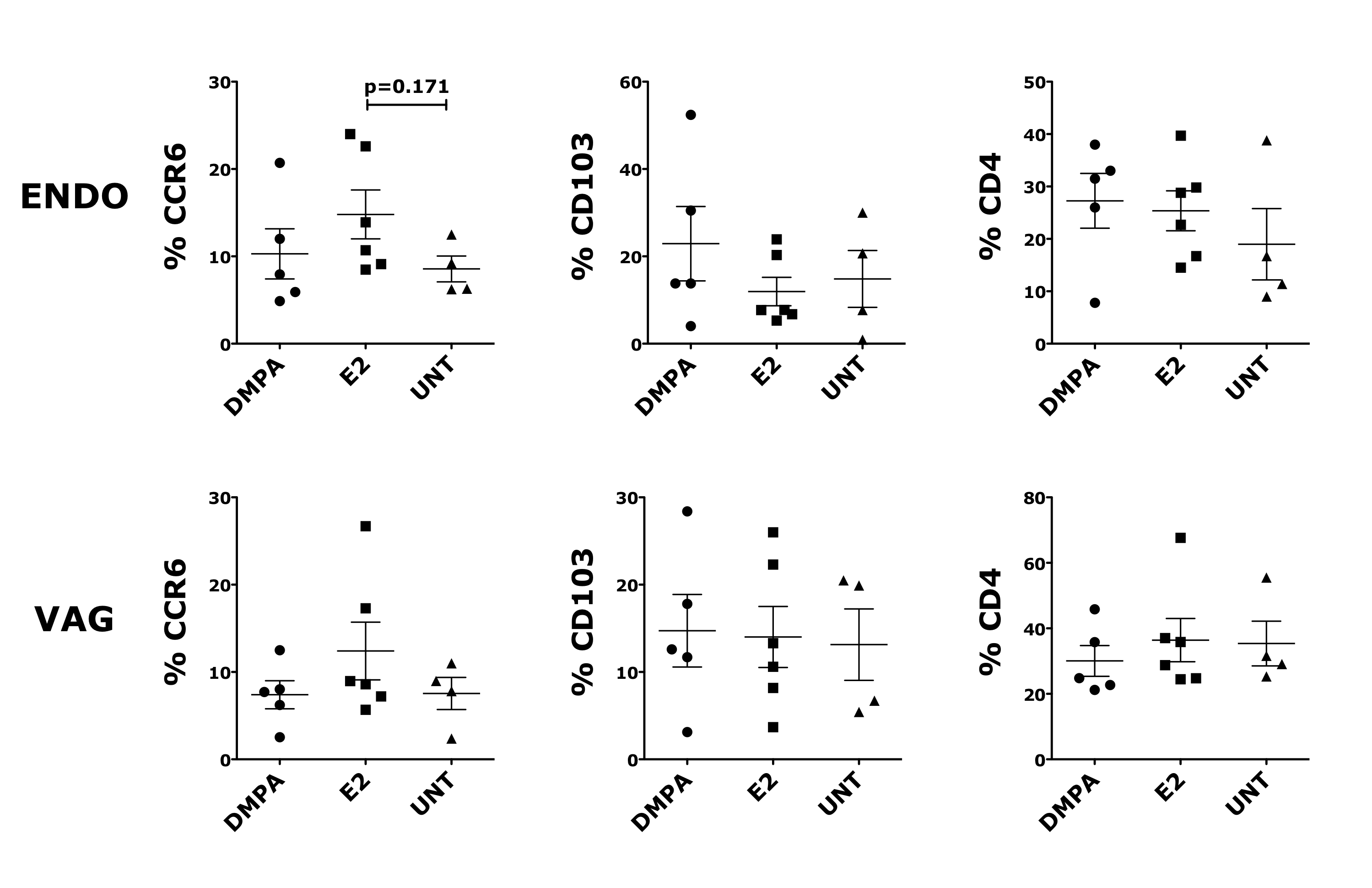

Supplement: Figure S2 — DMPA and E2 do not modulate the frequencies of CCR6+, CD103+ and CD4+ T cells in vaginal and endocervical tissues: Cells from endocervical and vaginal tissues were gated on live, singlets and on CD3+ CD4+ cells (left and center) or on CD3+ (right). The frequencies of CCR6+ and CD103+ cells within CD3+ CD4+ cells and the frequencies of CD4+ within CD3+ are shown. Bars represent mean±SEM. (TIF) [file pone.0097767.s002.tif]
