# Supplementary material for: Sex Hormones Selectively Impact the Endocervical Mucosal Microenvironment: Implications for HIV Transmission
Source: PLoS One. 2014 May 15;9(5):e97767. doi: 10.1371/journal.pone.0097767 (PMC4022654; doi:10.1371/journal.pone.0097767)
Supplement: Table S1 — List of primers used for the Sybr Green qPCR measuring the expression of the corresponding genes. (DOCX) [file pone.0097767.s003.docx]

**Supplemental Table 1**

| **Gene** | **FW 5’-3’** | **RV 5’-3’** | **Template** |
| --- | --- | --- | --- |
| GAPDH | GCCGCATTTTCTCTTGCATC | CTTCCCCATGGTGTCTCAGG | NM_001195426.1 |
| MAdCAM-1 | CTTGTGTACGCCTTCCCGGACCAGC | ACAGGCCACCTCCGGGTCAC | XR_091736.1 |
| CCR5 | GCACATTGCCAAACGCTTCT | AAACTGAACTTGCTCGCTCG | NM_001042773.2 |
| CCL21 | GCTGCCTCAAGTACAGCCAA | CGGGGCAAGAACAGGATAGC | NM_002989.3 |
| CCL4 | CAGCACCAATGGGCTCAG | CCTCGCGGTGTAAGAAAAGC | NM_001032873.1 |
| IFNα | TTGCTTTACTGGTGGCCCTG | GAGAGCAGCTTGACTTGCAGC | NM_001135794 |
| TGFβ | TGGAAACCCACAACGAAATCT | TGCTTCTCGGAGCTCTGATGT | XM_001100842.2 |
